# Biosynthesis, Antibacterial Activity and Anticancer Effects Against Prostate Cancer (PC-3) Cells of Silver Nanoparticles Using *Dimocarpus Longan Lour*. Peel Extract

**DOI:** 10.1186/s11671-016-1511-9

**Published:** 2016-06-17

**Authors:** Yan He, Zhiyun Du, Shijing Ma, Shupeng Cheng, Sen Jiang, Yue Liu, Dongli Li, Huarong Huang, Kun Zhang, Xi Zheng

**Affiliations:** Allan H. Conney Laboratory for Anticancer Research, School of Chemical Engineering and Light Industry, Guangdong University of Technology, Guangzhou, China; Susan Lehman Cullman Laboratory for Cancer Research, Department of Chemical Biology, Ernest Mario School of Pharmacy, Rutgers, The State University of New Jersey, 164 Frelinghusen Road, Piscataway, NJ 08854 USA

**Keywords:** Silver nanoparticles, *Dimocarpus Longan Lour.*, Bactericidal, Anticancer, Prostate cancer

## Abstract

Metal nanoparticles, particularly silver nanoparticles (AgNPs), are developing more important roles as diagnostic and therapeutic agents for cancers with the improvement of eco-friendly synthesis methods. This study demonstrates the biosynthesis, antibacterial activity, and anticancer effects of silver nanoparticles using *Dimocarpus Longan Lour.* peel aqueous extract. The AgNPs were characterized by UV-vis absorption spectroscopy, X-ray diffraction (XRD), high-resolution transmission electron microscopy (HRTEM), scanning electron microscopy (SEM), and Fourier transform infrared spectroscope (FTIR). The bactericidal properties of the synthesized AgNPs were observed via the agar dilution method and the growth inhibition test. The cytotoxicity effect was explored on human prostate cancer PC-3 cells in vitro by trypan blue assay. The expressions of phosphorylated stat 3, bcl-2, survivin, and caspase-3 were examined by Western blot analysis. The longan peel extract acted as a strong reducing and stabilizing agent during the synthesis. Water-soluble AgNPs of size 9–32 nm was gathered with a face-centered cubic structure. The AgNPs had potent bactericidal activities against gram-positive and gram-negative bacteria with a dose-related effect. AgNPs also showed dose-dependent cytotoxicity against PC-3 cells through a decrease of stat 3, bcl-2, and survivin, as well as an increase in caspase-3. These findings confirm the bactericidal properties and explored a potential anticancer application of AgNPs for prostate cancer therapy. Further research should be focused on the comprehensive study of molecular mechanism and in vivo effects on the prostate cancer.

## Background

Metal nanoparticles are most promising for their long use for medicinal purposes since ancient time and have attracted more attentions for potential applications in cancers [1, 2]. Among silver nanoparticles (AgNPs) are one of the significant members due to their unique physical-chemical properties, biological compatibility, relative lower toxicity, and biological activities such as antimicrobial, inflammatory, and anticancer activities [3–5].

AgNPs have been synthesized by various physical, chemical, electrochemical, photochemical, irradiative, and recently improved biological methods. Most of the ways are quite expensive or potentially dangerous to the environment [6]. While greener biological synthesis has better eco-friendly, cost-effective, and easily large-scaled properties and are motivating a research upsurge in exploring the plants as reducing and protecting agents [7], such as *Pulicaria glutinosa* [8], *P. glutinosa* [9], and *Moringa oleifera* [10]. Plant extracts are rich of functional molecules such as phenolic compounds, which have been regarded as potent natural reducers with high antioxidant activity [11, 12]. Longan (*Dimocarpus Longan Lour*.), an edible fruit, is cultivated widely in southern China and south-east Asia. With rich bioactive polysaccharides, phenolic acids, and flavonoids [13, 14], its flesh or seed is a good source of functional ingredients for antioxidant, immunity-modulatory, insomnia relief, and memory-enhancing effects [15–19]. Whereas peels, regarded as a waste usually, have an untapped potential in the resource utilization and might be available for potential green synthesis purposes.

The improvement of biosynthesized AgNPs has allowed better control of shape and size more suitable for the increasing biomedical applications, such as implantable biomaterials, tumor radiotherapy sensitization agent, molecular imaging agents, biological markers, and drug delivery [20, 21]. Silver is now extending its applications in cancer treatment as antitumor molecules, and many attempts turned up meaningful and positive [22, 23]. It was reported that AgNPs had antitumor effects against the cervical carcinoma cells [10], embryo fibroblast 3T3 cells [24], lung cancer H1299 cells [25], breast cancer MCF-7 cells [26], and glioblastoma multiforme U-87 cells [27]. AgNPs could be delivered into the cell by the Trojan effect and inhibit the RNA polymerase activity and the gene transcription via a direct reciprocal interaction [28]. The tumor cells were more sensitive to AgNPs damage than normal cells [29]. The particle size and surface features of AgNPs are very important for biomedical considerations. AgNPs with smaller particle size seemed to have a stronger penetration ability and greater toxicity for cancer cells [30]. The biochemical molecular mechanisms have not been fully revealed so far and more efforts should be tried.

Eco-friendly green synthesis with plant extracts to obtain ideal AgNPs for bactericidal and anticancer applications attracted our attentions. The present work investigated the synthesis of the AgNPs with a smaller size by peel extract of longan for the first time and further tested the antibacterial and in vitro anticancer effects against prostate cancer cells.

## Methods

### Materials

Silver nitrate (AgNO_3_) and 3-(4,5-dimethylthiazol-2-yl)-2,5-diphenyltetrazolium bromide (MTT) were sourced from Sigma-Aldrich (St Louis, MO, USA). Dried longan was obtained from Chinese Herbal Medicine Market in Guangzhou. *Escherichia coli* (ATCC25922), *Staphylococcus aureus* (ATCC 6538), *Bacillus subtilis* (ATCC6633), *Pseudomonas aeruginosa* (ATCC15442), and *Candida albicans* (ATCC10231) were offered by Guangdong Institute of Microbiology (Guangzhou, China). Prostate cancer PC-3 cells were from the ATCC (Rockville, MD, USA). RPMI-1640 medium, fetal bovine serum (FBS), L-glutamine, and penicillin-streptomycin were purchased from Gibco (Grand Island, NY, USA). Trypan blue stain was bought from Cambrex (Walkersville, MD, USA). Antibodies of β-actin, p-stat 3, survivin, and caspase-3 were purchased from Millipore Corporation (Billerica, MA, USA). Bcl-2 antibody and all secondary antibodies were provided by Santa Cruz Biotechnology (Dallas, TX, USA). Thermo assay kit with SuperSignal West Femto Luminol/Enhancer solution was from Thermo Scientific (Rockford, IL, USA).

### Synthesis of AgNPs

Longan peel was selected as the plant reducer for the synthesis of AgNPs. The peels were immersed in distilled water (10 g/250 mL) at 50 °C for 1 h. The extract filtrates were cooled and centrifuged at 3000 rpm for 5 min. Then, the filtrates were added drop by drop to AgNO_3_ (2 mM) aqueous solution at the ratio of 1:1. After the reaction at 80 °C for 5 h under stirring, the Ag colloid was then precipitated by the centrifugation (12,000 rpm, 20 min) and washed thrice with distilled water to make sure of the complete removal of extracts. The purified AgNPs were obtained after drying at 50 °C under vacuum oven for 24 h.

The effects of key factors such as (i) amount of longan peel extract, (ii) concentration of silver nitrate, and (iii) reaction temperature were explored to determine their effect on the synthesized nanoparticles. Different amounts of the aqueous extract (10, 20, 30, 40, and 50 mL) were added to a fixed concentration of AgNO_3_ (2.0 mM) at 80 °C. Different concentrations (0.5, 1.0, 2.0, 3.0, and 5.0 mM) of silver nitrate were also assessed when reacting with a 50 mL of the extract at 80 °C. Different reaction temperatures (room temperature and 80 °C) were explored with 50 mL of extract and 2.0 mM of silver nitrate. The experiment was continuously observed via color change with naked eye as well as UV-vis spectrophotometer [31].

### Characterization of AgNPs

The optimum AgNPs were further characterized by UV-vis absorption spectroscopy, X-ray diffraction (XRD), high-resolution transmission electron microscopy (HRTEM), scanning electron microscopy (SEM), and Fourier transform infrared spectroscope (FTIR). The stability of silver nanoparticles in aqueous solution was evaluated of the size distribution and plasmonic properties for 6 months at room temperature (RT) by TEM and UV-vis measurements. The optical property of synthesized AgNPs was observed by UV-vis double-beam spectrophotometer (UV-2450, Shimadzu, Kyoto, Japan) with a deuterium and tungsten iodine lamp in the range of 200–800 nm at RT. The size, shape, and surface morphology were under observation of HRTEM (H7100, Hitachi, Japan) and SEM (QUANTA400, FEI, Oregon, USA). The functional group and composition of AgNPs were characterized by FTIR (Nicolet 380, Thermo Electron, MA, USA) in the region of 4000–500 cm^−1^. Phase formation was analyzed XRD Analyzer (Ultima III, Rigaku, Tokyo, Japan) with Cu_kα_ radiation (*λ* = 1.5406 Å) in the 2*θ* range from 10° to 80° at the scanning rate of 4°/min. Mean crystallite diameter of the powder was determined by Debye-Scherrer equation from half width of diffraction peaks:

1 $$ D=\left(k\lambda \right)/\left(\beta\ \cos\ \theta \right) $$

where *D* is the mean crystallite diameter, *k* is a constant, *λ* is the wavelength of Cu_kα_, *θ* is the Bragg diffraction angle, and *β* is the full width at half-maximum.

### Antibacterical Activity of AgNPs

The bactericidal activities of AgNPs were tested against the gram-positive bacteria (*B. subtilis* and *S. aureus*), gram-negative bacteria (*E. coli* and *P. aeruginosa*), and the fungus (*C. albicans*), via agar dilution method and growth inhibition test assay [11]. For the agar dilution method, the 6-mm sterile plates were loaded with 20 μL of test samples of the biosynthesized AgNPs (2.0 mM), AgNO_3_ (2.0 mM), aqueous longan peel extract, or the penicillin solution (200 μg/mL). Each plate was inoculated with 1.0 × 10^7^ CFU of bacteria. Plates without AgNPs or with penicillin were used as a blank and positive control separately. The bacteria were cultured in Muller-Hinton agar (Luogang, Guangzhou, China) at 37 °C for 24 h and the fungus in Sabouraud’s dextrose agar (Luogang, Guangzhou, China) at 28 °C for 36 h. After incubation, the bactericidal activities were measured by the inhibition zones. For bacteria growth inhibition test, 100 μL of bacteria or fungus suspension with 1.0 × 10^**8**^ CFU were inoculated into 45 mL fresh LB media and treated series concentrations of AgNPs at 37 °C. The optical densities of the cultures were determined at 600 nm (OD600) by a UV-vis spectrophotometer.

### Cell Culture and Trypan Blue Exclusion Assay

PC-3 cells were cultured in RPMI-1640 medium with 10 % FBS, 100 units/mL streptomycin, 100 units/mL penicillin, and 300 μg/mL L-glutamine; 2 mL of cells were seeded at the density of 2 × 10^4^ cells/mL in the 35-mm dishes and cultured for 24 h at 37 °C in a humidified atmosphere of 5 % CO_2_. Then, the cells were exposed to different concentrations of AgNPs (2–30 μg/mL). After 72 h of treatment, 80 μL of the cell suspension was mixed with 20 μL of 0.4 % trypan blue stain solution for 2 min at RT. The cells were determined using a hemocytometer under a light microscope (Nikon Optiphot, Japan). The cells that did not absorb dye were regarded as live cells, and blue cells were counted as dead cells.

### Western Blot Analysis

The role of AgNPs for prostate cancer was further examined on the level of phosphorylated stat 3, bcl-2, survivin, and caspase-3 in PC-3 cells at the concentration of 10 μg/mL by Western blot according to our protocol [32, 33]. PC-3 cells were seeded at a density of 1 × 10^5^ cells/mL and incubated for 24 h. The cells were treated with 10 μg/mL of AgNPs. After 24 h treatment, the cells were washed with ice-cold PBS and lysed with 200 μL of lysis buffer (1 mM phenylmethylsulfonyl fluoride, 100 μM sodium orthovandate, 30 mM sodium pyrophosphate, 50 mM sodium chloride, 50 mM sodium fluoride, 5 mM ZnCl_2_, 2 mM iodoacetic acid, 10 mM Tris–HCl, and 0.5 % Triton X-100). Cell homogenates were centrifuged at 12,000*g* for 15 min at 4 °C. The protein concentrations were determined by the Bio-Rad protein assay kit. The β-actin protein was used as a loading control. Equal amounts of protein (50 μg) were loaded on a Bio-Rad Precast Gel (10 %) and then transferred to a PVDF membrane. The membranes were subsequently incubated with phosphor-stat 3, bcl-2, survivin, or caspase-3 primary antibodies (Millipore) at 4 °C overnight, respectively. At the end of incubation, all membranes were washed with 0.05 % Tween 20 Tris-buffered saline (TBST, 15 min) four times, then treated by the secondary antibody (Santa Cruz Biotechnology) for 1 h at RT. After washing the membranes again four times with TBST, immunoreactivity was detected by Thermo assay kit and analyzed by the Quantity One software (Bio-Rad).

## Results and Discussion

### Synthesis and Characterization of AgNPs

**Fig. 1:**
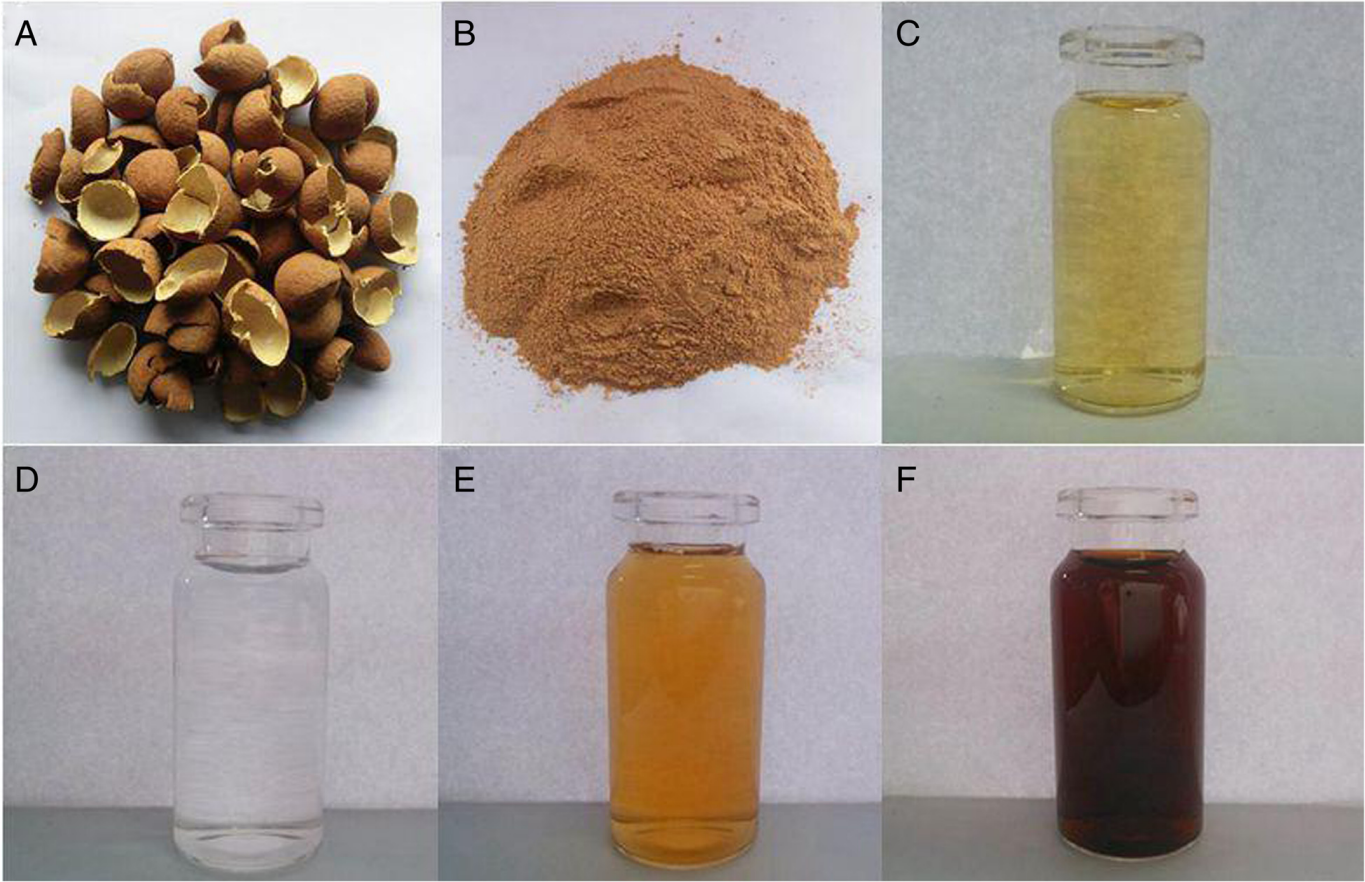
The photograph of longan peel (**a**) and the powder of longan peel (**b**), longan peel extracts (**c**), AgNO_3_ aqueous solution (**d**), reaction solution for 30 min (**e**), and the synthesized AgNPs solution (**f**)

**Fig. 2:**
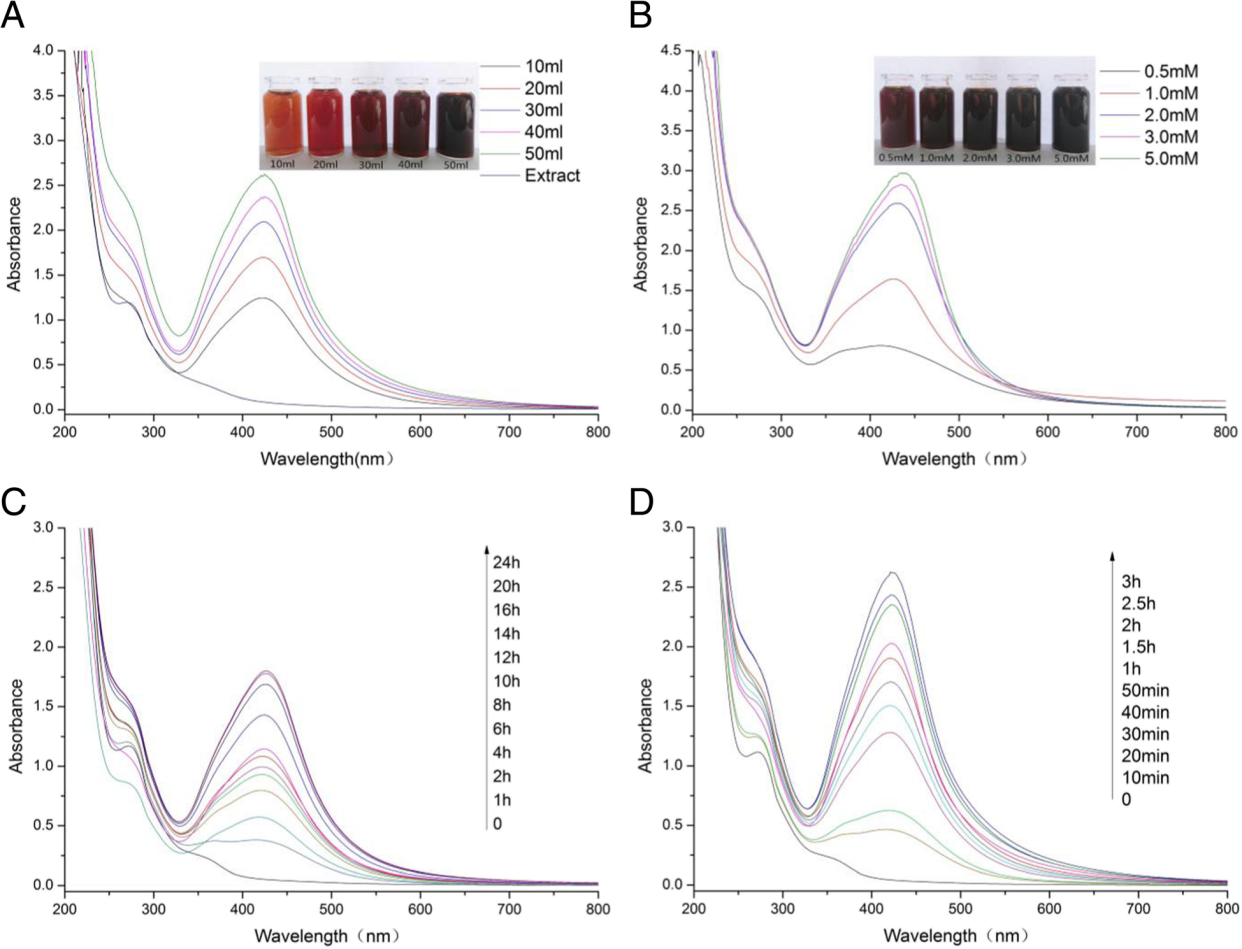
UV-vis absorption spectra of AgNPs during synthesis process, with various amount of extract at 80 °C for 3 h (**a**), with various concentrations of AgNO_3_ at 80 °C for 3 h (**b**), at RT (**c**), and at 80 °C (**d**)

**Fig. 3:**
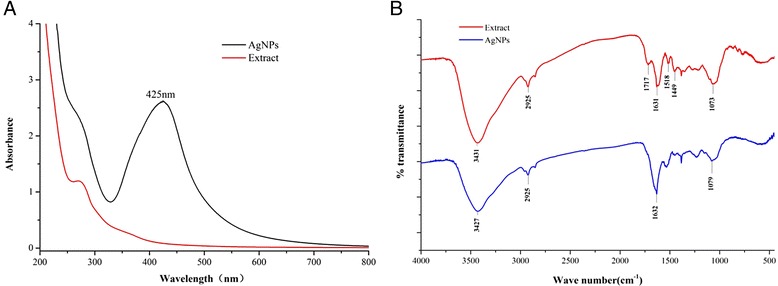
UV-vis absorption (**a**) and FTIR (**b**) spectrums of extracts and synthesized AgNPs

Eco-friendly green synthesis with plant extracts plays a very important role in nanotechnology without any harmful chemicals. Many natural plants extracts with a rich source of functional molecules has been verified as the capping and reducing agents for the synthesis of AgNPs [16]. We have before screened some edible plants, such as *Chrysanthemum morifolium* Ramat and *Shaddock* [11]. In this study, the synthesis reaction was started with the introduction of peel extract into aqueous silver nitrate solution. Silver nanoparticles exhibit yellowish brown color due to excitation of surface plasmon resonance (SPR) vibrations, so the color from colorless to dark brown confirmed the form of colloidal AgNPs from silver nitrate after incubation (Fig. 1). The AgNPs have the SFR absorption band with free electrons, due to the combined vibration of electrons of AgNPs in resonance with a light wave. AgNPs contribute to the absorption bands around 380–450 nm, among which there is no absorption for the extracts as shown in Fig. 3. The reduction of pure Ag^+^ ions to Ag^0^ could also be monitored by the UV-vis spectrum with the broad SFR at 425 nm (Figs. 2 and 3) [31].

We have obtained the well-synthesized AgNPs using longan extracts by exploring the effects of extract, silver nitrate, and reaction temperature. The reactions were observed under the UV-vis spectroscopy to examine the bioreduction of AgNPs from aqueous AgNO_3_ solution. Some small changes in synthetic factors lead to dramatic modifications in nanoparticle structure, size, morphology, stability, and self-assembly patterns [30], which could be indicated by the SPR band [34, 35]. The narrow peaks observed in the Fig. 2 give indirect evidence to the ability of the reductant to function as a stabilizer of the nanoparticles against aggregation. Figure 2a shows the effects of amount of plant extracts on the reaction process of AgNPs. No significant shift change of absorption peak appeared indicating no difference in the morphology and size of the formed silver particles. More stable samples could be collected with amount of the extract, while 50 mL above of the extract solutions could promote the agglomeration of nanosilver and stimulate the particle size larger. The spectra in Fig. 2b shows the influence of AgNO_3_ on the reaction. There was a wavelength shift following the concentration changes with absorption peak of 412, 427, 438, and 440 nm when reacted with 0.5, 1.0, 3, and 5.0 mM of AgNO_3_, respectively. The SPR wavelength shifts towards long-wavelength region which indicated larger particle size. The reaction with 20 mM and above of AgNO_3_ showed an obvious increase of yield. There are no significant difference between the peak absorption values between 20, 30, and 50 mM of AgNO_3._ To obtain the AgNPs with ideal size for the future investigation, 20 mM was finally decided for the synthesis. The UV-vis absorption spectrum of the obtained AgNPs was compared under room temperature (Fig. 2c) with 80 °C (Fig. 2d) at different time intervals. The process under 80 °C is more advantageous as it results in the formation of nanoparticles at a faster rate, higher yield, and slightly smaller size when compared with room temperature. Finally, we have developed an optimum green synthesis method for AgNPs by treating 2.0 mM AgNO_3_ with 50 mL of natural longan peel extract at 80 °C for 3 h without any harmful reducing, capping, or dispersing agents. The optimum AgNPs were further characterized and determined the antibacterial and anticancer effects.

Figure 3a shows the UV-vis absorption spectrum of the CM extract and synthesized AgNPs. A broad absorption peak was observed at 425 nm, which is a characteristic band for the Ag, arising from the excitation of longitudinal plasmon vibrations of AgNPs in the solution. FTIR measurements were performed to identify the potential biomolecules in longan peel extract responsible for capping and stabilizing the silver nanoparticles. The FTIR spectrum of silver nanoparticles in Fig. 3b showed very strong peak at 3427 cm^−1^ which is assigned as −OH stretching in alcohols and phenolic compounds [36]. The medium intense band 1632 cm^−1^ is assigned to the C=C aromatic vibrations [37]. The band at 2925 cm^−1^ ascribes to the C–H stretching vibration of methyl, methylene, and methoxy groups. The peak 1079 cm^−1^ indicates the presence of C–O stretching of alcohols, amide, ester, and ether groups. There represented some similar absorption peaks at 3431, 2925, 1631, and 1073 cm^−1^ in the extract spectrum. The peak at 1717 cm^−1^ could be attributed to the C=O aromatic vibrations. The broad band at 1518 and 1449 cm^−1^ assigned for the presence stretching vibrations of C=C in aromatic ring. The consistent vibrational bands such as –O–H, –C–H, –C=C, and –C=O are derived from the compounds such as flavonoids and terpenoids in longan peel. These functional groups may play an important role in the synthesis as the capping and reducing agent. Fully understanding of the underlying mechanism demands further exploration.

**Fig. 4:**
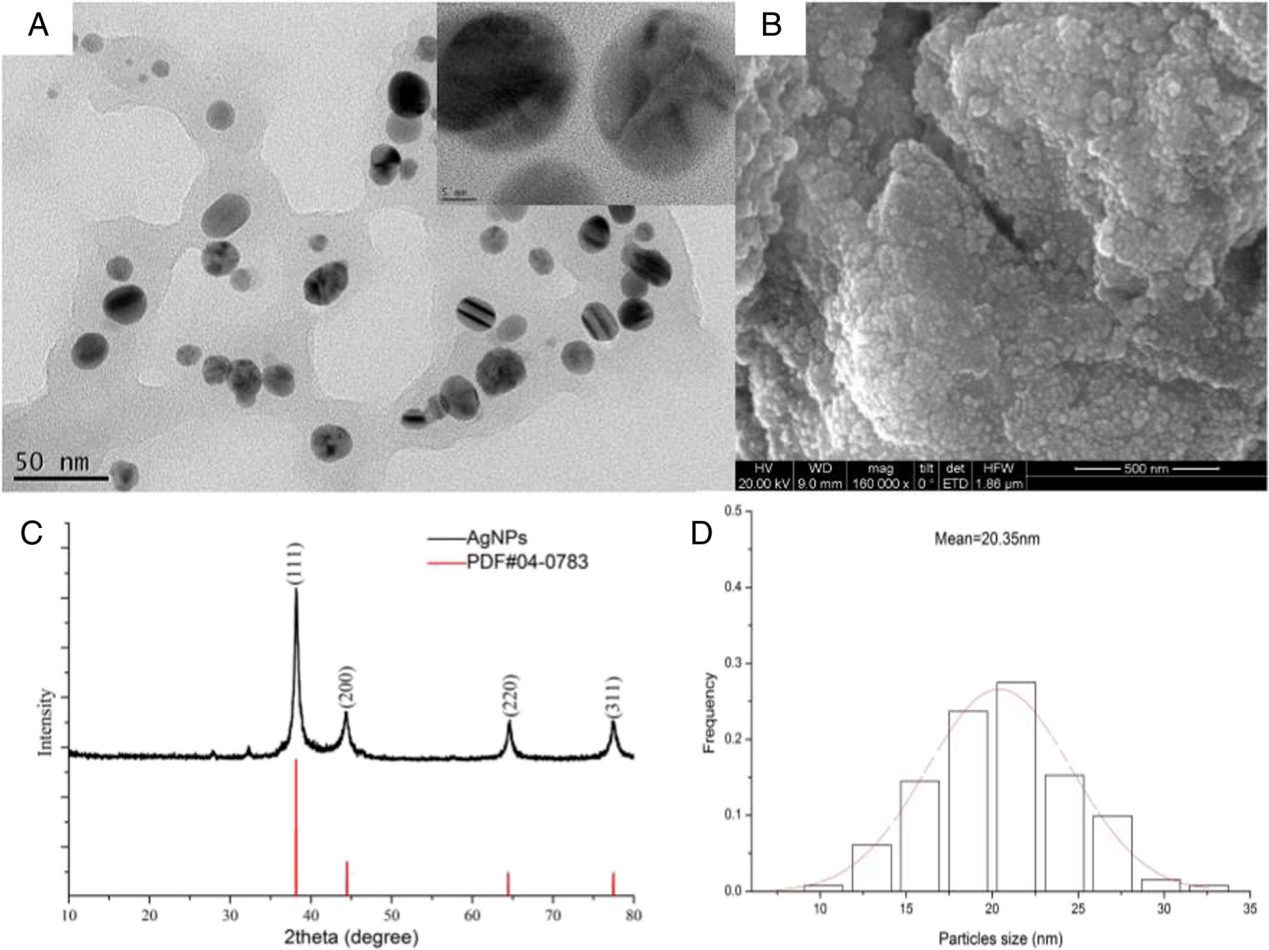
HRTEM images (**a**), SEM images (**b**), XRD images (**c**), and particle size distribution (**d**) of AgNPs

The morphology of the well-designed AgNPs was characterized by XRD, HRTEM, and SEM micrographs (Fig. 4). Figure 4c depicted four main characteristic diffraction peaks for Ag observed at 2*θ* of 38.2, 44.3, 64.4, and 77.6, which correspond to the (111), (200), (220), and (311) based on the band for face-centered cubic structures of silver, respectively (JCPDS Card No. 04-0783). No peaks from any other phase were observed showing that single-phase Ag with cubic structure nanoparticles has been obtained directly. The (111) plane was chosen to calculate crystalline size, and the average size of the synthesized AgNPs is 15 nm from the Debye-Scherrer equation. The HRTEM and SEM image presented that AgNPs were spherical and single crystalline with a narrow size range 9–32 nm and the average size 20.35 nm, which is in good agreement with the shape of the SPR band in the UV-vis spectrum and the particle size calculated from XRD analysis. The well-controlled AgNPs, especially their sizes, is of critical importance to the activities and toxicity [38]. The AgNPs with smaller particle size have a stronger penetration ability and greater toxicity [39]. The cancer cells are different in pore size compared to other cells, and so a size-controlled targeting of AgNPs can prove effective in the case of cancer treatment [34]. Our synthesized AgNPs was spherical with such a size which is wildly recognized among a good range for anticancer drug delivery applications and medical purposes [40].

### Stability of AgNPs in Aqueous Solution

**Fig. 5:**
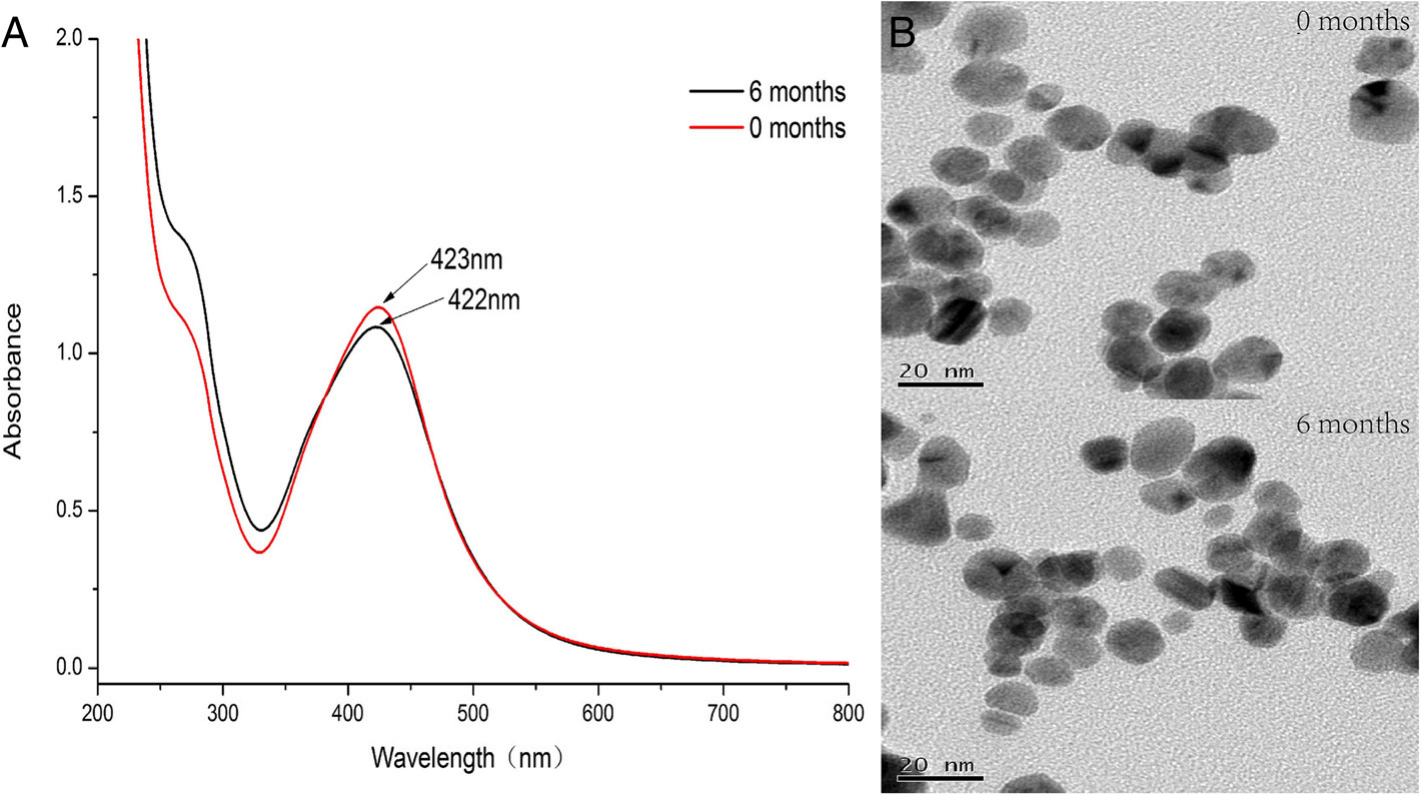
Stability of AgNPs in aqueous solution for 6 months at RT. Photographs of **a** UV-vis and **b** TEM images

As to the stability of silver nanoparticles in aqueous solution, we evaluated the size distribution and plasmonic properties for 6 months at RT by TEM and UV-vis measurements (Fig. 5). It was found from TEM images that AgNPs are well-dispersed without any aggregation, as well as with no significant change in particle size and shape. There was also stable in plasmonic properties, indicating good stability of AgNPs for 6 months. This is corresponding to the previous study [41]. Good stability of nano-silver in many researches is discussed under different standard cell incubation conditions [42, 43]. Foldbjerg et al. studied the cytotoxicity and genotoxicity of silver nanoparticles in the human lung cancer A549 cell, which indicated that the size and stability of the AgNPs remain the same in DMEM and RPMI also used as the exposure medium in our study [44].

### Antimicrobial Activity of AgNPs

**Fig. 6:**
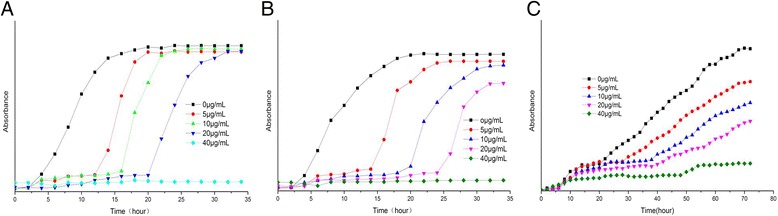
Growth curves of *E. coli* (**a**), *S. aureus* (**b**) and *C. albicans* (**c**) in LB media with different concentrations of AgNPs

As indicated in Table 1, the inhibition zones of AgNPs were 15, 20, 16, 19, and 13 mm for *S. aureus*, *B. subtilis*, *E. coli*, *P. aeruginosa*, and *C. albicans*, respectively. The extract tested had no bactericidal activity. The synthesized AgNPs shows a higher inhibitory effect compared with AgNO_3_ and could compete commercial antimicrobial agents penicillin used for the treatment of bacterial infections. Figure 6 shows the good growth inhibition of AgNPs on *E. coli* and *S. aureus.* The growth of *E. coli* was completely inhibited in 35 h at 100, 24 h at 50, and 16 h at 20 μg/mL. Meanwhile, the growth of *S. aureus* was totally suppressed in 35 h at 100, 20 h at 50, and 16 h at 20 μg/mL. AgNPs had better effects on bacteria than fungi, as well as greater impacts on gram-negative bacteria than gram-positive bacteria in this study, which is corresponded with the previous researches [45]. The strong bactericidal activity of silver nanoparticles is verified because they can easily enter the pathogen with a unique small size and surface effects [46]. The cell membrane of the bacteria might be destroyed due to the reaction of amino (−NH), −SH, −COOH, and other functional groups in the cell [47]. AgNPs could also depress the synthesis of bacterial metabolism enzyme, destroy the genetic factor, and eventually lead to death [48]. The presence of peptidoglycan layer in the cell wall of gram-positive bacteria could attribute to less activity, and it can prevent the nanoparticles from penetrating into bacteria [49].

**Table 1:** Antibacterial activity of the biosynthesized AgNPs

Microorganisms	Zone of inhibition(mm in diameter)			
	Penicillin	AgNPs	AgNO_3_	Extract
*S. aureus*	24	15	10	–
*B. subtilis*	23	20	12	–
*E. coli*	21	16	10	–
*P. aeruginosa*	25	19	9	–
*C. albicans*	16	13	8	–

### Anticancer Effects of AgNPs on PC-3 Cells

**Fig. 7:**
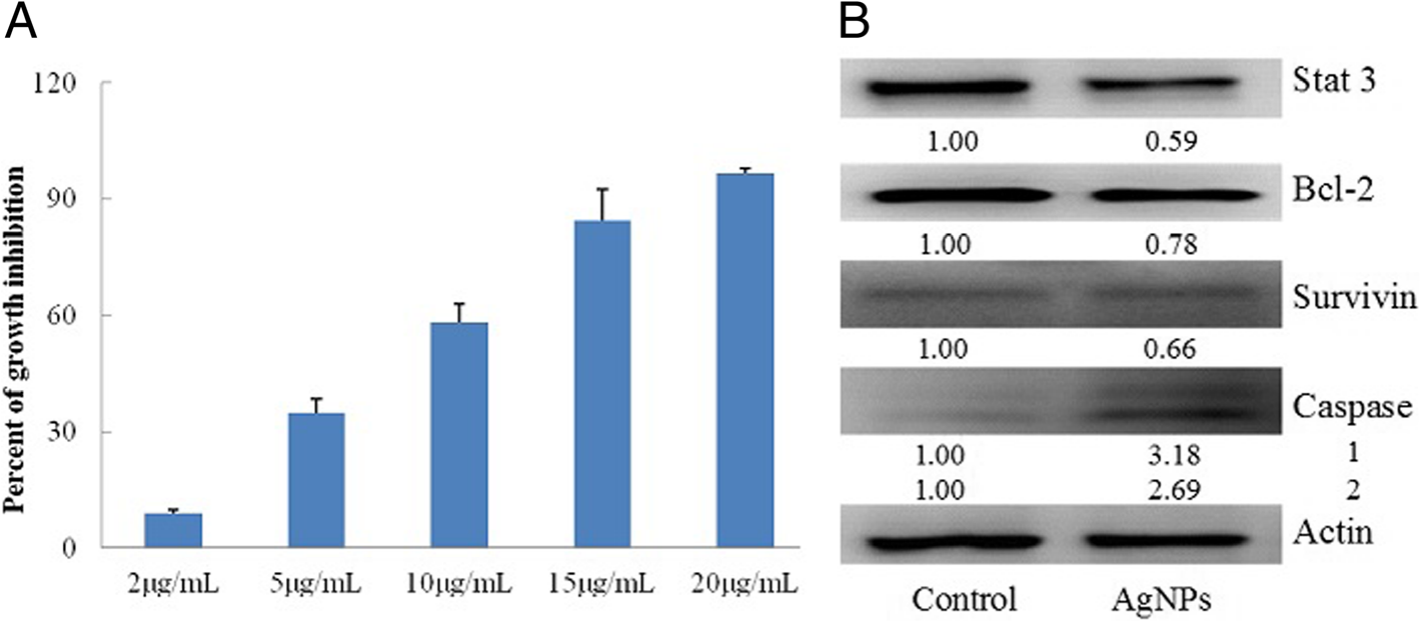
The anticancer effects of AgNPs on the PC-3 cells, **a** cytotoxic effects and **b** effects of the level of phosphorylated stat 3, bcl-2, survivin, and caspase-3

Cancers figure among the leading causes of morbidity and mortality worldwide, with approximately 14 million new cases and 8.2 million cancer-related deaths in 2012 [50]. Prostate cancer is the second most common cancer and the fifth leading cause of cancer death in men in 2012 [51]. AgNPs have been shown to have important bactericidal and antiangiogenic activities, which are attractive for the research of their potent anticancer effects. The effect and the underlying mechanism of our AgNPs with a smaller size range on prostate cancer attracted our curiosity. In this study, the cytotoxicity was evaluated on PC-3 cells in vitro by the trypan blue exclusion assay. As shown in Fig. 7a, the synthesized AgNPs of size 9–32 nm had dose-dependent cytotoxic effects on prostate cancer PC-3 cells in vitro*.* About 50 % of PC-3 cells died when treated with AgNPs at the concentrations between 5 and 10 μg/mL. The IC_50_ was less than 10 μg/mL, which is consistent with the previous findings [52]. The protein expressions were explored by Western blot to clarify the mechanism by which AgNPs exert a cytotoxic effect on PC-3 cells. The antitumor activities were mediated by a decrease of stat 3, bcl-2, and survivin, as well as an increase in caspase-3 shown in Fig. 7b.

The cytotoxic effect of AgNPs on cell viability has a major role in antitumor activity, thereby reducing disease progression. Cellular internalization of silver might provide the basis for the cytotoxicity of AgNPs [39, 53]. Sriram et al. found that AgNPs could enter the cell through endocytosis, acted on the nucleus to interfere with chromosome stability, and interrupt the mitosis [29]. Active anticancer components are known to exhibit a consistent antitumor effect by blocking abnormal expression alterations of multiple signaling proteins, such as VEGF, p53, JAK-STAT, and CDC14A [45, 54, 55]. The antitumor activity of AgNPs was also shown to be mediated by induction of proliferation and apoptosis by active caspase 9 and active caspase 3 in a GBM tumor system in vitro [56]. These biological responses eventually lead to the survival or apoptosis of the cancer cells and prompt a promising way of AgNPs for prostate cancer. The molecular mechanism of targeting has not fully understood for the inhibitory effects of AgNPs on the prostate cancer cells. Further research efforts are deserved to elucidate the latent mechanism and then lead to more effective measures for prostate cancer.

## Conclusions

With better developments of the green synthesis methods and understanding of the molecular mechanism, the applications of AgNPs are likely to expand further in the cancer therapy fields. In the present work, we have proposed first time a simple, high efficiency, eco-friendly green synthesis method for AgNPs using longan peel extract as reducing and stabilizing agent demonstrated. The biosynthezied AgNPs are spherical and single crystalline, with a narrow particle size range from 9 to 32 nm. The synthesized AgNPs showed good bactericidal activities against gram-positive and gram-negative bacteria and fungus with a dose-dependent effect. AgNPs exhibited a strong inhibitory effect on the prostatic cancer PC-3 cells, which might be associated with a decrease of stat 3, bcl-2, and survivin, as well as an increase in caspase-3. AgNPs might be a potential active candidate for prostate cancer treatments. Further research should be focused on the comprehensive study of molecular mechanism and in vivo effects of AgNPs on prostate cancer.

## References

[1] Rai M, Ingle AP, Gupta I, Brandelli A (2015). Bioactivity of noble metal nanoparticles decorated with biopolymers and their application in drug delivery. Int J Pharm.

[2] Sharma H, Mishra PK, Talegaonkar S, Vaidya B (2015). Metal nanoparticles: a theranostic nanotool against cancer. Drug Discov Today.

[3] Austin LA, Mackey MA, Dreaden EC, El-Sayed MA (2014). The optical, photothermal, and facile surface chemical properties of gold and silver nanoparticles in biodiagnostics, therapy, and drug delivery. Arch Toxicol.

[4] Dos Santos CA, Seckler MM, Ingle AP, Gupta I, Galdiero S, Galdiero M, Gade A, Rai M (2014). Silver nanoparticles: therapeutical uses, toxicity, and safety issues. J Pharm Sci.

[5] Majdalawieh A, Kanan MC, El-Kadri O, Kanan SM (2014). Recent advances in gold and silver nanoparticles: synthesis and applications. J Nanosci Nanotechnol.

[6] Wei L, Lu J, Xu H, Patel A, Chen ZS, Chen G (2015). Silver nanoparticles: synthesis, properties, and therapeutic applications. Drug Discov Today.

[7] Chung IM, Park I, Seung-Hyun K, Thiruvengadam M, Rajakumar G (2016). Plant-mediated synthesis of silver nanoparticles: their characteristic properties and therapeutic applications. Nanoscale Res Lett.

[8] Khan M, Khan M, Adil SF, Tahir MN, Tremel W, Alkhathlan HZ, Al-Warthan A, Siddiqui MR (2013). Green synthesis of silver nanoparticles mediated by Pulicaria glutinosa extract. Int J Nanomedicine.

[9] Khan M, Khan ST, Khan M, Adil SF, Musarrat J, Al-Khedhairy AA, Al-Warthan A, Siddiqui MR, Alkhathlan HZ (2014). Antibacterial properties of silver nanoparticles synthesized using Pulicaria glutinosa plant extract as a green bioreductant. Int J Nanomedicine.

[10] Vasanth K, Ilango K, MohanKumar R, Agrawal A, Dubey GP (2014). Anticancer activity of Moringa oleifera mediated silver nanoparticles on human cervical carcinoma cells by apoptosis induction. Colloids Surf B: Biointerfaces.

[11] He Y, Du Z, Lv H, Jia Q, Tang Z, Zheng X, Zhang K, Zhao F (2013). Green synthesis of silver nanoparticles by Chrysanthemum morifolium Ramat. extract and their application in clinical ultrasound gel. Int J Nanomed.

[12] Harshiny M, Matheswaran M, Arthanareeswaran G, Kumaran S, Rajasree S (2015). Enhancement of antibacterial properties of silver nanoparticles-ceftriaxone conjugate through Mukia maderaspatana leaf extract mediated synthesis. Ecotoxicol Environ Saf.

[13] Chen J, Ge ZZ, Zhu W, Xu Z, Li CM (2014). Screening of key antioxidant compounds of longan (Dimocarpus Longan Lour.) seed extract by combining online fishing/knockout, activity evaluation, Fourier transform ion cyclotron resonance mass spectrometry, and high-performance liquid chromatography electrospray ionization mass spectrometry methods. J Agric Food Chem.

[14] Rangkadilok N, Worasuttayangkurn L, Bennett RN, Satayavivad J (2005). Identification and quantification of polyphenolic compounds in longan (Euphoria Longana Lam.) fruit. J Agric Food Chem.

[15] Jiang YM, Zhang ZQ, Joyce DC, Ketsa S (2002). Postharvest biology and handling of longan fruit (Dimocarpus Longan Lour.). Postharvest Biol Technol.

[16] Prasad KN, Yang B, Shi J, Yu C, Zhao M, Xue S, Jiang Y (2010). Enhanced antioxidant and antityrosinase activities of longan fruit pericarp by ultra-high-pressure-assisted extraction. J Pharm Biomed Anal.

[17] Zhong K, Wang Q, He Y, He X (2010). Evaluation of radicals scavenging, immunity-modulatory and antitumor activities of longan polysaccharides with ultrasonic extraction on in S180 tumor mice models. Int J Biol MacroMol.

[18] Park SJ, Park DH, Kim DH, Lee S, Yoon BH, Jung WY, Lee KT, Cheong JH, Ryu JH (2010). The memory enhancing effects of Euphoria longan fruit extract in mice. J Ethnopharmacol.

[19] Yang B, Jiang YM, Shi J, Chen F, Ashraf M (2011). Extraction and pharmacological properties of bioactive compounds from longan (Dimocarpus Longan Lour.) fruit—a review. Food Res Int.

[20] Chaloupka K, Malam Y, Seifalian AM (2010). Nanosilver as a new generation of nanoproduct in biomedical applications. Trends Biotechnol.

[21] Stensberg MC, Wei Q, McLamore ES, Porterfield DM, Wei A, Sepúlveda MS (2011). Toxicological studies on silver nanoparticles: challenges and opportunities in assessment, monitoring and imaging. Nanomedicine.

[22] Ong C, Lim JZ, Ng CT, Li JJ, Yung LY, Bay BH (2013). Silver nanoparticles in cancer: therapeutic efficacy and toxicity. Curr Med Chem.

[23] Lima R, Seabra AB, Durán N (2012). Silver nanoparticles: a brief review of cytotoxicity and genotoxicity of chemically and biogenically synthesized nanoparticles. J Appl Toxicol.

[24] Arokiyaraj S, Arasu MV, Vincent S, Prakash NU, Choi SH, Oh YK, Choi KC, Kim KH (2014). Rapid green synthesis of silver nanoparticles from Chrysanthemum indicum L and its antibacterial and cytotoxic effects: an in vitro study. Int J Nanomedicine.

[25] Gengan RM, Anand K, Phulukdaree A, Chuturgoon A (2013). H1299 lung cell line activity of biosynthesized silver nanoparticles using Albizia adianthifolia leaf. Colloids Surf B: Biointerfaces.

[26] Farah MA, Ali MA, Chen SM, Li Y, Al-Hemaid FM, Abou-Tarboush FM, Al-Anazi KM, Lee J (2016). Silver nanoparticles synthesized from Adenium obesum leaf extract induced DNA damage, apoptosis and autophagy via generation of reactive oxygen species. Colloids Surf B: Biointerfaces.

[27] Locatelli E, Naddaka M, Uboldi C, Loudos G, Fragogeorgi E, Molinari V, Pucci A, Tsotakos T, Psimadas D, Ponti J, Franchini MC. Targeted delivery of silver nanoparticles and alisertib: in vitro and in vivo synergistic effect against glioblastoma. Nanomedicine (Lond). 2014;9(6):839-49. 10.2217/nnm.14.1 24433240

[28] Wang Z, Liu S, Ma J, Qu G, Wang X, Yu S, He J, Liu J, Xia T, Jiang GB (2013). Silver nanoparticles induced RNA polymerase-silver binding and RNA transcription inhibition in erythroid progenitor cells. ACS Nano.

[29] Sriram MI, Kanth SB, Kalishwaralal K, Gurunathan S (2010). Antitumor activity of silver nanoparticles in Dalton’s lymphoma ascites tumor model. Int J Nanomedicine.

[30] Li L, Sun J, Li X, Zhang Y, Wang Z, Wang C, Dai J, Wang Q (2012). Controllable synthesis of monodispersed silver nanoparticles as standards for quantitative assessment of their cytotoxicity. Biomaterials.

[31] Shivshankar S, Ahmad A, Rai A, Sastry M (2004). Rapid synthesis of Au, Ag and bimetallic Au core-Ag shell nanoparticles by using neem (Azadirachta indica) leaf broth. J Colloid Interface Sci.

[32] Huang H, He Y, Cui XX, Goodin S, Wang H, Du ZY, Li D, Zhang K, Tony Kong AN, DiPaola RS, Yang CS, Conney AH, Zheng X (2014). Potent inhibitory effect of δ-tocopherol on prostate cancer cells cultured in vitro and grown as xenograft tumors in vivo. J Agric Food Chem.

[33] Dongli Li DZ, Li C, Liu Y, Goodin S, Huang H, He Y, Zhang Y, Wang H, Zheng X, Zhang K (2015). Potent inhibitory effect of terpenoids from Acanthopanax trifoliatus on growth of PC-3 prostate cancer cells in vitro and in vivo is associated with suppression of NF-kB and STAT3 signalling. J Funct Foods.

[34] Amendola V, Bakr OM, Stellacci F (2010). Study of the surface plasmon resonance of silver nanoparticles by the discrete dipole approximation method: effect of shape, size, structure, and assembly. Plasmonics.

[35] Smitha SL, Nissamuddin KM, Philip D, Gopchandran KG (2008). Studies on surface plasmon resonance and photoluminescence of silver nanoparticles. Spectrochim Acta A Mol BioMol Spectrosc.

[36] Kasturi J, Veerapandian S, Rajendiran N (2009). Biological synthesis of silver nanoparticles using apiin as reducing agent. Colloid Surf B: Biointerfaces.

[37] Subarani S, Sabhanayakam S, Kamaraj C. Studies on the impact of biosynthesized silver nanoparticles (AgNPs) in relation to malaria and filariasis vector control against Anopheles stephensi Liston and Culex quinquefasciatus Say (Diptera: Culicidae). Parasitol Res. 2013;112(2):487-99. 10.1007/s00436-012-3158-5 23064800

[38] Park MV, Neigh AM, Vermeulen JP, de la Fonteyne LJ, Verharen HW, Briedé JJ, van Loveren H, de Jong WH (2011). The effect of particle size on the cytotoxicity, inflammation, developmental toxicity and genotoxicity of silver nanoparticles. Biomaterials.

[39] Carlson C, Hussain SM, Schrand AM, Braydich-Stolle LK, Hess KL, Jones RL, Schlager JJ (2008). Unique cellular interaction of silver nanoparticles: size-dependent generation of reactive oxygen species. J Phys Chem B.

[40] Albanese A, Tang PS, Chan WC (2012). The effect of nanoparticle size, shape, and surface chemistry on biological systems. Ann Rev Biomed Eng.

[41] Muniyappan N, Nagarajan NS (2014). Green synthesis of silver nanoparticles with *Dalbergia spinosa* leaves and their applications in biological and catalytic activities [J]. Process Biochem.

[42] Reidy B, Haase A, Luch A, Dawson KA, Lynch I (2013). Mechanisms of silver nanoparticle release, transformation and toxicity: a critical review of current knowledge and recommendations for future studies and applications. Materials.

[43] Hansen U, Thünemann AF (2015). Characterization of silver nanoparticles in cell culture medium containing fetal bovine serum. Langmuir.

[44] Foldbjerg R, Dang DA, Autrup H (2011). Cytotoxicity and genotoxicity of silver nanoparticles in the human lung cancer cell line, a549. Arch Toxicol.

[45] Fayaz AM, Balaji K, Girilal M, Yadav R, Kalaichelvan PT, Venketesan R (2010). Biogenic synthesis of silver nanoparticles and their synergistic effect with antibiotics: a study against gram-positive and gram-negative bacteria. Nanomedicine.

[46] Li WR, Xie XB, Shi QS, Zeng HY, Ou-Yang YS, Chen YB (2010). Antibacterial activity and mechanism of silver nanoparticles on Escherichia coli. Appl MicroBiol Biotechnol.

[47] Priyadarshini S, Gopinath V, Meera PN, MubarakAli D, Velusamy P (2013). Synthesis of anisotropic silver nanoparticles using novel strain, Bacillus flexus and its biomedical application. Colloids Surf B: Biointerfaces.

[48] Rai M, Yadav A, Gade A (2009). Silver nanoparticles as a new generation of antimicrobials. Biotechnol Adv.

[49] Xu L, Li X, Takemura T, Hanagata N, Wu G, Chou LL (2012). Genotoxicity and molecular response of silver nanoparticle (NP)-based hydrogel. J Nanobiotechnology.

[50] Stewart BW, Wild CP (2014). World cancer report 2014.

[51] Ouvinha de Oliveira R, de Santa Maria LC, Barratt G (2014). Nanomedicine and its applications to the treatment of prostate cancer. Ann Pharm Fr.

[52] Firdhouse MJ, Lalitha P (2013). Biosynthesis of silver nanoparticles using the extract of Alternanthera sessilis—antiproliferative effect against prostate cancer cells. Cancer Nanotechnol.

[53] Söderstjerna E, Bauer P, Cedervall T, Abdshill H, Johansson F, Johansson UE (2014). Silver and gold nanoparticles exposure to in vitro cultured retina—studies on nanoparticle internalization, apoptosis, oxidative stress, glial- and microglial activity. PLoS One.

[54] Sheikpranbabu S, Kalishwaralal K, Venkataraman D, Eom SH, Park J, Gurunathan S (2009). Silver nanoparticles inhibit VEGF-and IL-1-induced vascular permeability via Src dependent pathway in porcine retinal endothelial cells. J Nanobiotechnology.

[55] Satapathy SR, Mohapatra P, Preet R, Das D, Sarkar B, Choudhuri T, Wyatt MD, Kundu CN (2013). Silver-based nanoparticles induce apoptosis in human colon cancer cells mediated through p53. Nanomedicine.

[56] Urbańska K, Pająk B, Orzechowski A, Sokołowska J, Grodzik M, Sawosz E, Szmidt M, Sysa P (2015). The effect of silver nanoparticles (AgNPs) on proliferation and apoptosis of in ovo cultured glioblastoma multiforme (GBM) cells. Nanoscale Res Lett.

